# Biofilm microbiome in extracorporeal membrane oxygenator catheters

**DOI:** 10.1371/journal.pone.0257449

**Published:** 2021-09-16

**Authors:** Yeuni Yu, Yun Hak Kim, Woo Hyun Cho, Bong Soo Son, Hye Ju Yeo

**Affiliations:** 1 Interdisciplinary Program of Genomic Science, Pusan National University, Yagnsan, Republic of Korea; 2 Department of Anatomy and Department of Biomedical Informatics, School of Medicine, Pusan National University, Yangsan, Republic of Korea; 3 Division of Pulmonary, Allergy, and Critical Care Medicine, Department of Internal Medicine, Pusan National University Yangsan Hospital, Yagnsan, Republic of Korea; 4 Department of Thoracic and Cardiovascular Surgery, Pusan National University Yangsan Hospital, Yangsan, Republic of Korea; 5 Research Institute for Convergence of Biomedical Science and Technology, Pusan National University Yangsan Hospital, Yagnsan, Republic of Korea; Universidade Federal do Rio de Janeiro, BRAZIL

## Abstract

Despite the formation of biofilms on catheters for extracorporeal membrane oxygenation (ECMO), some patients do not show bacteremia. To elucidate the specific linkage between biofilms and bacteremia in patients with ECMO, an improved understanding of the microbial community within catheter biofilms is necessary. Hence, we aimed to evaluate the biofilm microbiome of ECMO catheters from adults with (n = 6) and without (n = 15) bacteremia. The microbiomes of the catheter biofilms were evaluated by profiling the V3 and V4 regions of bacterial 16s rRNA genes using the Illumina MiSeq sequencing platform. In total, 2,548,172 reads, with an average of 121,341 reads per sample, were generated. Although alpha diversity was slightly higher in the non-bacteremic group, the difference was not statistically significant. In addition, there was no difference in beta diversity between the two groups. We found 367 different genera, of which 8 were present in all samples regardless of group; *Limnohabitans*, *Flavobacterium*, *Delftia*, *Massilia*, *Bacillus*, *Candidatus*, *Xiphinematobacter*, *and CL0-1* showed an abundance of more than 1% in the sample. In particular, *Arthrobacter*, *SMB53*, *Neisseria*, *Ortrobactrum*, *Candidatus Rhabdochlamydia*, *Deefgae*, *Dyella*, *Paracoccus*, and *Pedobacter* were highly abundant in the bacteremic group. Network analysis indicated that the microbiome of the bacteremic group was more complex than that of the non-bacteremic group. *Flavobacterium* and *CL0*.*1*, which were abundant in the bacteremic group, were considered important genera because they connected different subnetworks. Biofilm characteristics in ECMO catheters varied according to the presence or absence of bacteremia. There were no significant differences in diversity between the two groups, but there were significant differences in the community composition of the biofilms. The biofilm-associated community was dynamic, with the bacteremic group showing very complex network connections within the microbiome.

## Introduction

Bacteria adapt to life on catheter surfaces through a number of metabolic changes, including the production of extracellular substances and the regulation of specific genes [1]. They form networks, termed biofilms, which enable multicellular functions. Bacterial biofilms provide a beneficial survival system for community members [2]. Biofilms on intravascular catheters are the most common cause of nosocomial septicemia and catheter-related bloodstream infections (CRBSI) [3]. Despite efforts to maintain sterility, catheters can easily become contaminated with bacteria. Biofilms present a significant challenge in terms of their sampling, diagnosis, and treatment to prevent infection [4].

Extracorporeal membrane oxygenation (ECMO) requires vascular cannulation to support critically ill patients. Cannulas are maintained for several days to weeks depending on the patient’s condition, and exchanging cannulas is almost impossible. Blood stream infections (BSIs) are significant complications of ECMO and are associated with mortality, morbidity, and increased healthcare costs in ECMO patients [5]. Bacteremia during ECMO has been associated with catheter colonization, and in a previous study, we found biofilm-related infections in ECMO catheters. However, despite the formation of biofilms on the catheters, some patients did not develop bacteremia [6], and it is unclear why this did not occur. A deeper understanding of the microbial community within catheter biofilms is necessary to elucidate the specific linkage between biofilms and bacteremia. Catheter microbiomes may play an important role in the development of bacteremia from biofilms. To determine the microbiome characteristics related to biofilms in patients who developed bacteremia from ECMO catheters, we compared the differences in the catheter biofilm microbiome between patients with and without bacteremia.

## Materials and methods

### Study design and clinical examination

This single-center, retrospective cohort study was conducted using prospectively collected ECMO catheter specimens. Patients aged > 18 years who received ECMO support between November 2016 and April 2017 were included. The catheter specimens were collected from patients who provided written consent and were stored in the Pusan National University Yangsan Hospital Biobank. The biobank provided samples and clinical data collected from consented patients. We retrospectively compared the clinical and microbiome data between patients with (n = 6) and those without (n = 15) bacteremia. This study was approved by the Institutional Review Board of Pusan National University Yangsan Hospital (No. 05-2020-146).

### Sample collection and preparation

ECMO catheters were aseptically collected from patients upon removal at the completion of the ECMO treatment. Sections of the catheter that had been within the intravascular space (5 cm) were cut, split longitudinally, transported immediately to the laboratory for standard catheter culture as mentioned in a previous study, and sent to the biobank for 16s rRNA sequencing [6]. Bacterial culture and catheter identification were conducted as follows. Catheter pieces were rolled across the surfaces of standard 5% sheep blood agar plates and MacConkey agar plates immediately after collection. After roll-plating, both plates were incubated for 48 h at 35°C in a 5% CO_2_/air atmosphere. An automated VITEK 2 system (BioMérieux, St. Louis, MO, USA) was used to identify isolates and for susceptibility testing. Clinical breakpoints recommended by the Clinical and Laboratory Standards Institute were used to define susceptibility and resistance [7]. Methicillin-resistant *Staphylococcus epidermidis* was defined as an *S*. *epidermidis* isolate with a minimum inhibitory concentration (MIC) ≥ 0.5 μg/mL for oxacillin. Carbapenem-resistant *Acinetobacter baumannii* was defined as an *A*. *baumannii* isolate with an MIC ≥ 16 μg/ml for imipenem. Extended spectrum β-lactamase (ESBL)-positive *Escherichia coli* was defined as an *E*. *coli* resistant to cefotaxime (MIC ≥ 0.5 μg/mL) and/or ceftazidime (MIC ≥ 0.5 μg/mL) and/or cefepime (MIC ≥ 1 μg/mL), which was inhibited by clavulanic acid.

### Extraction of total genomic DNA

DNA was extracted from ECMO catheter samples using a DNA purification kit (Lucigen Biosearch Technology, Novato, CA, USA) following the manufacturer’s instructions. The final concentration was measured with a NanoDrop ND-1000 spectrophotometer (Thermo Fisher Scientific, USA) and stored at -80°C until use.

### Polymerase chain reaction (PCR) amplification and sequencing analysis

Each sequenced sample was prepared according to Illumina 16S Metagenomic Sequencing Library protocols. Quantification of DNA and DNA quality was performed using PicoGreen and NanoDrop (Thermo Fisher Scientific, Wilmington, USA). The 16S rRNA genes were amplified using 16S V3-V4 primers, with the following primer sequences: 16S amplicon PCR forward primer (TCGTCGGCAGCGTCAGATGTGTATAAGAGACAGCCTACGGGNGGCWGCAG16S) and Amplicon PCR Reverse Primer (GTCTCGTGGGCTCGGAGATGTGTATAAGAGACAGGACTACHVGGGTATCTAATCC).

Input genomic DNA was amplified with 16S V3-V4 primers and a subsequent limited cycle amplification step was performed to add multiplexing indices and Illumina sequencing adapters. The final products were normalized and pooled using PicoGreen, and the size of the libraries was verified using a TapeStation DNA Screentape D1000 (Agilent, Waldbronn, Germany). Sequencing was performed using the MiSeq™ platform (Illumina, San Diego, CA, USA).

Raw reads were processed with QIIME2 v.2019.10 using the DADA2 plugin to decrease noise in the quality filter reads, call amplicon sequence variants (ASVs), and generate a feature table of ASV counts and host metadata [8]. In the quality filtering step, datasets were truncated to a read length of 265 to 280 base pairs for the forward and reverse reads, and a chimera removal step was performed. Following quality filtering, bacterial taxonomies were assigned to the ASV feature table using the naïve Bayesian Q2 classifier feature in QIIME2. The data obtained were compared to the Greengenes reference sequence database of the V3-V4 region of the 16S rRNA gene [9].

### Bioinformatic analysis, statistical analysis, and visualization

To evaluate alpha diversity, sample microbiota were estimated using Chao1 and Shannon indices. The Wilcoxon rank-sum test was used to compare significant differences in the alpha diversity indices between the different groups (*P* < 0.05). The similarity of the microbial community structure among all samples was evaluated using principal coordinates analysis (PCoA) at the operational taxonomic unit (OTU) level. An analysis of similarities was calculated to compare the intra- and inter-group similarities based on Bray-Curtis dissimilarity, which was calculated using QIIME2.

### LEfSe and PICRUSt analyses

Linear discriminant analysis (LDA) effect size (LEfSe) analyses were performed on the samples [10]. LDA was performed to determine the features (taxa) differentially represented between bacteremia and non-bacteremia samples. LEfSe combines the Kruskal-Wallis test or pairwise Wilcoxon rank-sum test with LDA. It ranks features by effect size, ranking features that explain more of the biological differences higher. The cut-off value was an absolute LDA score (log_10_) of > 2.5. The functional capacity of ECMO catheter microbiota was predicted using the Phylogenetic Investigation of Communities by Reconstruction of Unobserved States (PICRUSt) (Galaxy Version 1.0.0) [11]. The closed reference OTU table was generated from quality control reads in QIIME2 against the Greengenes reference sequence database. The closed OTU table drawn by QIIME2 was compared with the Kyoto Encyclopedia of Genes and Genomes (KEGG) database to obtain functional predictions. PICRUSt predictions were categorized as levels 1–3, relative to the KEGG pathways.

### Network analysis

Sparse Correlations for Compositional data (SparCC) was developed to calculate correlations between OTU frequencies in the microbiome data while accounting for their inherent sparseness and composition [12]. The microbiome network was constructed using SparCC to identify the links involved in co-abundance with an absolute pairwise correlation of > 0.7. The resulting correlations among taxa were graphed as a network using the *igraph* package [13] in R and visualized in Cytoscape [14].

## Results

### Patient characteristics and clinical outcomes

Patient characteristics and clinical outcomes are summarized in Table 1. The median age was 55 years (inter quartile range, IQR: 47–62.5 y), 71.4% (n = 15) of the patients were male, and the median ECMO duration was 9 days (IQR 6–14.5 d). There were no significant differences in patient characteristics between the two groups except for the APACHE II scores (median 13 vs. 10, *P* = 0.019) and the percentage of pneumonia (66.7% vs. 20%, *P* = 0.040) was higher in the bacteremic group. There were no significant differences in clinical outcomes.

**Table 1:** Patient characteristics.

Characteristic	Bacteremic (n = 6)	Non-bacteremic (n = 15)	*P*
**Age**	56.5 (46.3–62.5)	55 (47–63)	0.668
**Male**	4 (66.7)	11 (73.3)	0.760
**BMI**	25.5 (21.7–26.4)	22.8 (20.4–27.9)	0.697
**SOFA**	14 (10.3–16.3)	12 (10–15)	0.531
**APACHE II**	13 (11.8–18)	10 (8–12)	0.019
**Vasopressor**	5 (83.3)	11 (73.3)	0.627
**RRT**	2 (33.3)	4 (26.7)	0.760
**Pre ECMO PF ratio**	62.5 (56.5–68.5)	66 (57–81)	0.640
**ECMO duration (d)**	12 (7.8–23.5)	9 (4–11)	0.227
**ICU day**	21.5 (15.1–48.0)	42.0 (19.5–70.9)	0.436
**Pneumonia**	4 (66.7)	3 (20)	0.040
**Antibiotic use**	6 (100)	15 (100)	1.000
**ECMO complications**			
**Bleeding**	2 (33.3)	1 (6.7)	0.115
**Thrombosis**	0	1 (6.7)	0.517
**Weaning success from ECMO**	5 (83.3)	14 (93.3)	0.481
**Survival to discharge**	5 (83.3)	14 (93.3)	0.481

Data are presented as median (interquartile range) or n (%). BMI, body mass index; SOFA, sequential organ failure assessment; APACHE II, Acute Physiology and Chronic Health Evaluation II; RRT, renal replacement therapy; ECMO, extracorporeal membrane oxygenation; PF ratio, PaO_2_/FiO_2_; ICU, intensive care unit.

In the bacteremic group, the most common organism isolated by blood culture within 3 d of catheter removal was methicillin-resistant *S*. *epidermidis* (4/6, 66.7%), followed by carbapenem-resistant *A*. *baumannii* (1/6, 16.7%), and ESBL-positive *E*. *coli* (1/6, 16.7%).

### Diversity of bacterial community in ECMO

**Fig 1:**
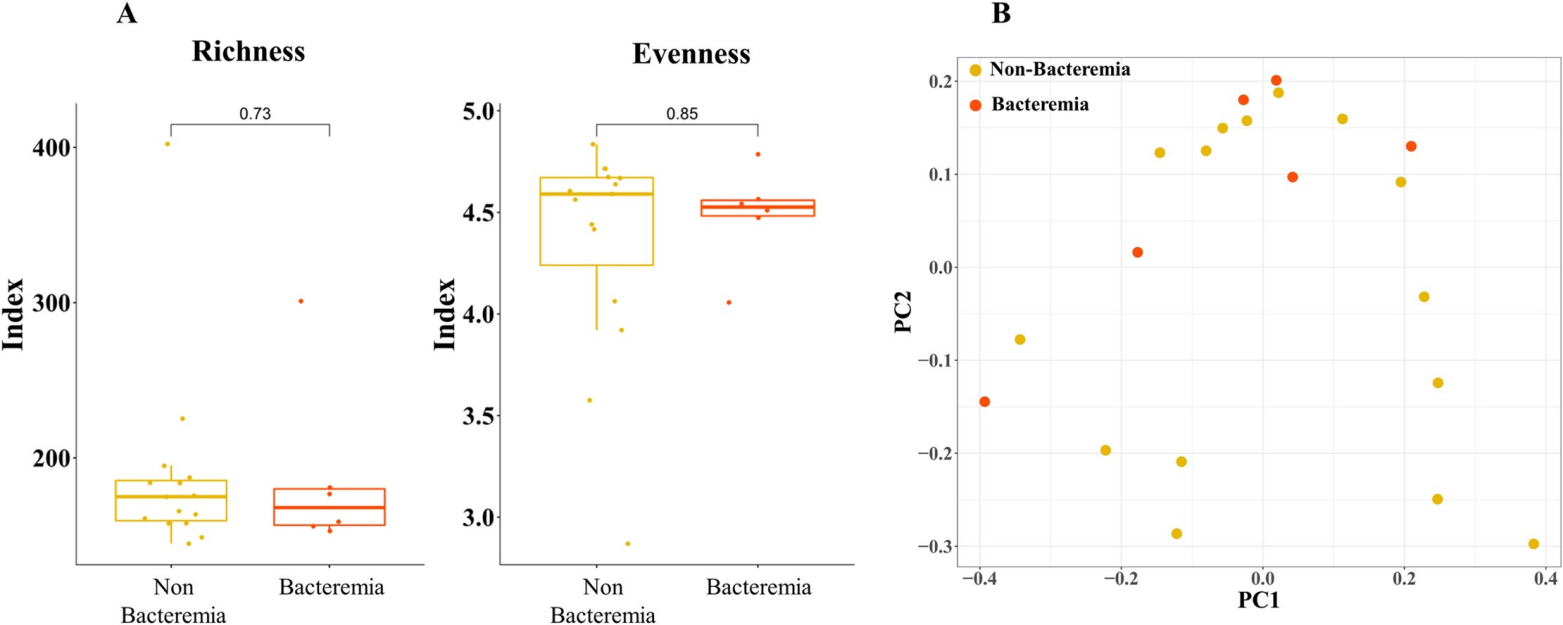
Taxonomic diversity of ECMO microbiomes. (A) Comparison of alpha diversity indices between bacteremic and non-bacteremic groups. (B) Principal coordinates analysis plot based on Bray-Curtis dissimilarity.

In total, 2,548,172 reads, with an average of 121,341 reads per sample, were generated after initial quality filtering and chimera removal (S1 Table). Initially, we measured the alpha diversity of the bacterial communities in each group. Total bacterial diversity was estimated using the Chao1 index. The evenness of the microbiota was estimated using the Shannon index (Fig 1A). Although it was slightly higher in the non-bacteremic group, there was no statistically significant difference in the Wilcoxon rank-sum test.

To examine the microbial community variability between the two groups, we calculated beta diversity by decomposing microbiome variability onto major components using PCoA on Bray-Curtis dissimilarity. (Fig 1B) There were no significant differences between the two groups.

### Differences in microbiome composition according to the presence of bacteremia

**Fig 2:**
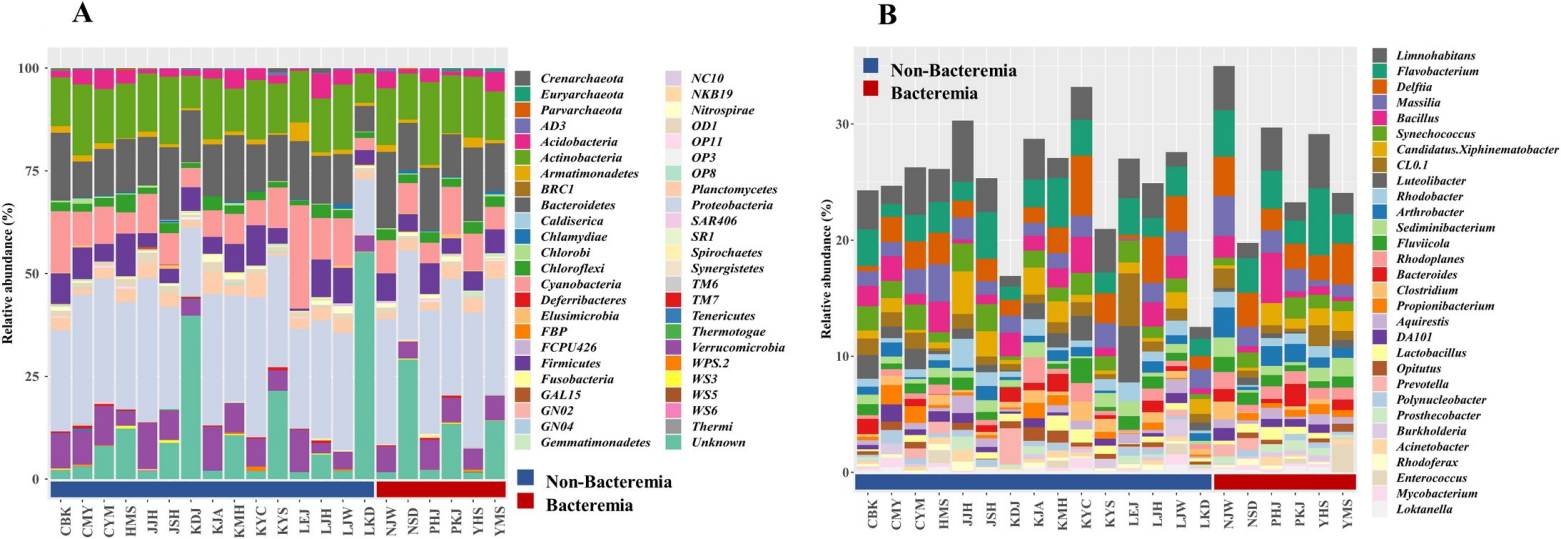
Taxonomic profiles of extracorporeal membrane oxygenation samples. (A) Phylum level taxonomic profile. (B) Genus level taxonomic profile.

To identify the microbiome profiles of the two groups, we examined the taxonomic composition and the relative abundance of bacteremia at different taxonomic levels. At the phylum level, we found 47 different phyla, 11 of which were present in all the samples. The major phyla were *Proteobacteria*, *Actinobacteria*, *Bacteroidetes*, *Cyanobacteria*, *Verrucomicrobia*, and *Firmicutes*, each of which formed more than 10% of the sample (Fig 2A).

We found 367 different genera, of which eight were present in all samples, regardless of group. Among them, *Limnohabitans*, *Flavobacterium*, *Delftia*, *Massilia*, *Bacillus*, *Candidatus*, *Xiphinematobacter*, and *CL0-1* showed an abundance of more than 1% in the sample. Upon comparing with the average, *Delftia* was slightly higher in the non-bacteremic group. *Bacillus*, *Flavobacterium*, *CL0-1*, and *Candidatus Xiphinematobacter* were more abundant in the bacteremic group. However, this difference was not statistically significant (Fig 2B).

### Difference in dominant microbiota between the groups

**Fig 3:**
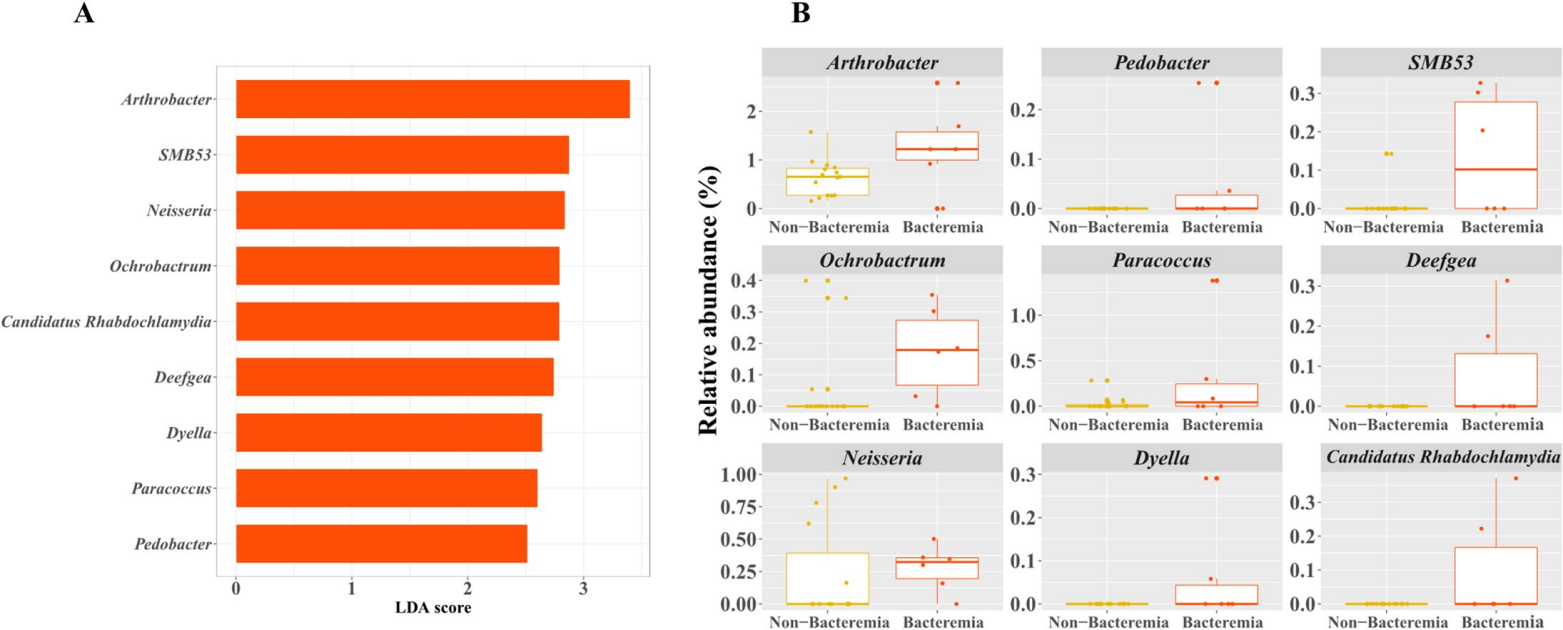
Differential abundance of taxa between bacteremic and non-bacteremic groups. (A) Taxa identified by linear effect size with linear discriminant analysis (LDA) values of 2.5. Taxa enriched in different groups are displayed by color indicated in the key (red indicates taxa abundant in bacteremia) (B). Relative abundance of the nine discriminative genera selected from the LDA results.

We used LEfSe to determine the taxa that most likely explained the differences between the two groups. In this study, a *P* value < 0.05, and an LDA score > 2.5 were considered to be significant. Differentially abundant taxa and their relative abundances are shown in Fig 3. Significant differences were found between the two groups. LEfSe analysis revealed nine discriminative genera, all of which were more abundant in the bacteremic group (Fig 3A): *Arthrobacter*, *SMB53*, *Neisseria*, *Ochrobactrum*, *Candidatus Rhabdochlamydia*, *Deefgea*, *Dyella*, *Paracoccus*, and *Pedobacter* (Fig 3B).

### Network analysis of the ECMO microbiome

**Fig 4:**
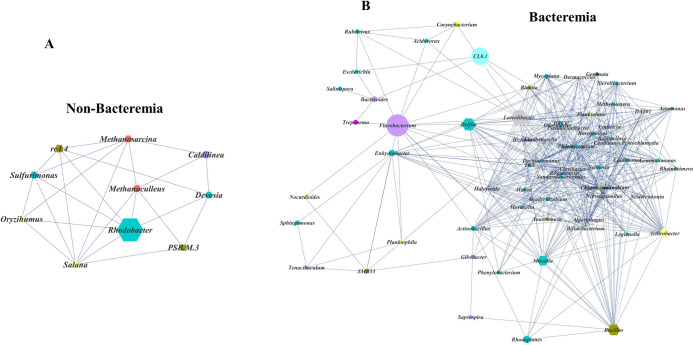
Identification and comparison of highly connected clusters of co-occurring networks of extracorporeal membrane oxygenation microbiota. (A) Non-bacteremic group. (B) Bacteremic group. Each node represents a genus and is colored by its assigned phylum. Hexagonal nodes represent hub taxa.

To determine the relationship between taxa associated with the bacteremic state, we conducted a network analysis at the genus level. The bacteremic group was very complex compared to the patients without bacteremia. In non-bacteremic patients, only genera associated with the hub were correlated (Fig 4A). However, the microbiome of bacteremic patients showed a high correlation with genera that were not associated with the hub (Fig 4B). Although not a hub, *Flavobacterium* and *CL0*.*1*, which were abundant in the bacteremic group, were considered important genera because they connected different subnetworks.

### Metabolic prediction

**Fig 5:**
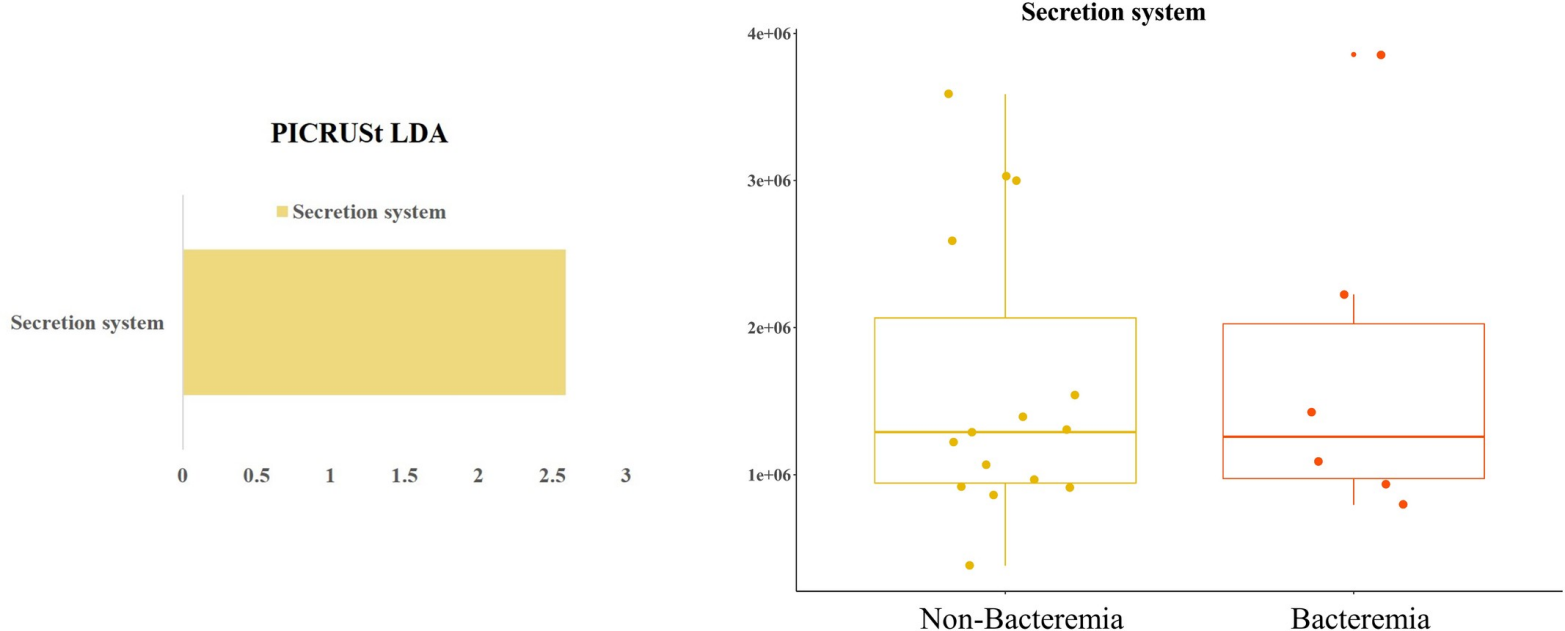
Differentially abundant gene functions between the non-bacteremic and bacteremic groups in the biofilm microbiome in extracorporeal membrane oxygenator catheters (ECMO). (A) Gene functions enriched in the non-bacteremic group are colored green. The box plot represents the predicted value of the secretion system. Functional categories of genes on the ECMO catheters were predicted using Phylogenetic Investigation of Communities by Reconstruction of Unobserved States. Differentially abundant functions were identified using linear discriminant analysis coupled with effect size measurements.

We explored whether metabolic pathways in the bacteremic state were related to the metabolic differences found between the two groups. We performed a comparative prediction analysis of the functional metagenome using PICRUSt. Among the 328 affiliated KEGG pathways, only one was shown to be statistically significant (*P* < 0.05; and LDA > 2). Although the LDA results indicated that the secretion system was significantly increased in the non-bacteremic group, the distribution of the secretion system value predicted using PICRUSt was not different between the two groups (Fig 5).

## Discussion

We detected significant differences in the microbiota of biofilms on ECMO catheters depending on the presence or absence of bacteremia. There was no significant difference in the microbial diversity. However, the dominant microbiota differed significantly between the two groups. Additionally, compared to the non-bacteremic group, the bacteremic group exhibited a very complex network connection within its microbiome.

To our knowledge, there have been no prior studies on the association between ECMO catheter microbiomes, biofilms, and bacteremia. In microbial communities, many microorganisms exhibit a synergistic relationship and depend on each other for survival [15]. The diversity and composition of microbial communities within biofilms may contribute to biofilm dispersal and BSI [16]. In this study, there were significant differences in the microbiota of the biofilm depending on the presence of bacteremia. Although there was no significant difference in diversity between the two groups, there was a difference in the dominant microbiota between the two groups. At the genus level, *Delftia* was more abundant in the non-bacteremic group, but *Bacillus*, *Flavobacterium*, *CL0-1*, *Candidatus Xiphinematobacter* were more abundant in the bacteremic group. This suggests that differences in the composition of the microbial community within the biofilm may be more important than its diversity in the development of BSI.

In this study, there was no correlation between the dominant microbiota and actual bacteremic pathogens. Previously, *Bacillus* and *Flavobacterium* have been reported as causes of device-related BSI in patients with significant underlying conditions [17–20]. *Arthrobacter*, *SMB53*, *Dyella*, *Paracoccus*, and *Pedobacter* were not identified in traditional cultures, but their DNA was detected on medical devices in patients with infection and they were postulated to be the cause of medical device-related infections [21–25]. Currently, the significance of the presence of other bacteria, including *CL0-1*, *Xiphinematobacter*, *Ortrobactrum*, *Candidatus Rhabdochlamydia*, and *Deefgae*, for biofilm formation and bacteremia is not clear. *Flavobacterium* and *CL0*.*1* demonstrated a connection with different subnetworks, highlighting the possibility of an important genus in the development of bacteremia. Further research is required on the role of these bacteria in bacteremia and biofilm production.

## Conclusion

In conclusion, there were significant differences in the biofilm microbiota of ECMO catheters based on the presence of bacteremia. Each ECMO catheter had biofilms composed of diverse bacterial communities. There were no significant differences in diversity between the two groups, but there were significant differences in the community composition of the biofilms. The biofilm-associated community was dynamic, with the bacteremic group showing highly complex network connections within the microbiome. Evidence for such a relationship is still limited, but these results may provide a critical background for studying the link between biofilms and bacteremia. Further research is required on the bacterial community within biofilms to elucidate biofilm-associated infections.

## Supporting information

S1 Table Analysis of biofilms in extracorporeal membrane oxygenation catheters. (DOCX)

S1 File Data sets. (ZIP)
