# Correction: Biofilm microbiome in extracorporeal membrane oxygenator catheters

**DOI:** 10.1371/journal.pone.0315755

**Published:** 2024-12-10

**Authors:** Yeuni Yu, Yun Hak Kim, Woo Hyun Cho, Bong Soo Son, Hye Ju Yeo

There are errors in the author affiliations. The correct affiliations are as follows:

Yeuni Yu^1^, Yun Hak Kim^2^, Woo Hyun Cho^3^, Bong Soo Son^4^, Hye Ju Yeo^3,5^

1 Interdisciplinary Program of Genomic Science, Pusan National University, Yagnsan, Republic of Korea, 2 Department of Anatomy and Department of Biomedical Informatics, School of Medicine, Pusan National University, Yangsan, Republic of Korea, 3 Division of Pulmonary, Allergy, and Critical Care Medicine, Department of Internal Medicine, Pusan National University Yangsan Hospital, Pusan National University School of Medicine, Yagnsan, Republic of Korea, 4 Department of Thoracic and Cardiovascular Surgery, Pusan National University Yangsan Hospital, Pusan National University School of Medicine, Yangsan, Republic of Korea, 5 Research Institute for Convergence of Biomedical Science and Technology, Pusan National University Yangsan Hospital, Pusan National University School of Medicine, Yagnsan, Republic of Korea.

## References

[1] Yu Y, Kim YH, Cho WH, Son BS, Yeo HJ (2021) Biofilm microbiome in extracorporeal membrane oxygenator catheters. PLOS ONE 16(9): e0257449. doi: 10.1371/journal.pone.0257449 34529734 PMC8445415

