# Supplementary material for: Biofilm microbiome in extracorporeal membrane oxygenator catheters
Source: PLoS One. 2021 Sep 16;16(9):e0257449. doi: 10.1371/journal.pone.0257449 (PMC8445415; doi:10.1371/journal.pone.0257449)
Supplement: S1 Table — (DOCX) [file pone.0257449.s001.docx]

Supplementary Table1. Analysis of biofilms in extracorporeal membrane oxygenation catheters

| **Sample** | **Input** | **Filtered** | **Denoised** | **Merged** | **Non-chimeric** | **Phylum** | **Genus** |
| --- | --- | --- | --- | --- | --- | --- | --- |
| Non-bacteremia | |  |  |  |  |  |  |
| CBK | 403524 | 283711 | 281032 | 256248 | 228824 | 31 | 142 |
| CMY | 243341 | 172653 | 171310 | 166381 | 162297 | 21 | 68 |
| CYM | 360656 | 241370 | 238229 | 225628 | 221541 | 16 | 77 |
| HMS | 151911 | 91256 | 87463 | 77199 | 75621 | 19 | 73 |
| JJH | 151639 | 108937 | 108315 | 102361 | 92945 | 18 | 101 |
| JSH | 153859 | 99547 | 96235 | 87831 | 86356 | 19 | 73 |
| KDJ | 316534 | 170585 | 158169 | 120658 | 119130 | 20 | 80 |
| KJA | 143057 | 101103 | 100709 | 98200 | 94306 | 13 | 74 |
| KMH | 120492 | 75755 | 72820 | 64521 | 62556 | 17 | 73 |
| KYC | 126109 | 88616 | 88331 | 86162 | 84712 | 12 | 68 |
| KYS | 160109 | 92978 | 87607 | 72817 | 71733 | 15 | 53 |
| LEJ | 464535 | 334845 | 331768 | 300345 | 244764 | 24 | 111 |
| LJH | 124528 | 83608 | 81738 | 77275 | 75366 | 17 | 73 |
| LJW | 130132 | 92239 | 91895 | 89898 | 88758 | 16 | 69 |
| LKD | 174470 | 92204 | 82396 | 58692 | 58045 | 15 | 60 |
| Bacteremia | |  |  |  |  |  |  |
| NJW | 129449 | 93684 | 93426 | 91385 | 89801 | 14 | 60 |
| NSD | 193640 | 111372 | 104425 | 82796 | 81102 | 19 | 63 |
| PHJ | 113150 | 81078 | 80841 | 79147 | 76557 | 16 | 56 |
| PKJ | 366198 | 228723 | 222186 | 199907 | 195972 | 21 | 71 |
| YHS | 168378 | 118042 | 117314 | 114693 | 113603 | 15 | 71 |
| YMS | 393457 | 256265 | 251006 | 230775 | 224183 | 17 | 68 |
| Sum | 4589168 | 3018571 | 2947215 | 2682919 | 2548172 | 375 | 1584 |
| Median | 160109 | 101103 | 100709 | 91385 | 89801 | 17 | 71 |
| Mean | 218532 | 143741 | 140344 | 127758 | 121342 | 18 | 75 |
